# Pro-inflammatory gene expression in solid glioblastoma microenvironment and in hypoxic stem cells from human glioblastoma

**DOI:** 10.1186/1742-2094-8-32

**Published:** 2011-04-13

**Authors:** Marco Tafani, Maura Di Vito, Alessandro Frati, Laura Pellegrini, Elena De Santis, Giovanni Sette, Adriana Eramo, Patrizio Sale, Emanuela Mari, Antonio Santoro, Antonino Raco, Maurizio Salvati, Ruggero De Maria, Matteo A Russo

**Affiliations:** 1 Department of Experimental Medicine, Sapienza University of Rome, Italy; 2 Department of Neurosurgery, IRCCS Neuromed, Pozzilli, Italy; 3 Department of Human Anatomy, Sapienza University of Rome, Italy; 4 Laboratory of Haematology and Oncology, ISS, Istituto Superiore di Sanità, Rome, Italy; 5 Department of Cellular and Molecular Pathology, IRCCS San Raffaele Pisana, Rome, Italy; 6 Department of Neurology and Psychiatry, Sapienza University of Rome, Italy

## Abstract

**Background:**

Adaptation to hypoxia and consequent pro-inflammatory gene expression of prostate and breast carcinomas have been implicated in the progression toward cancer malignant phenotype. Only partial data are available for the human tumor glioblastoma multiforme (GBM). The aim of our study was to analyze the hypoxic and pro-inflammatory microenvironment in GBMs and to demonstrate that in a stem/progenitor cell line derived from human glioblastoma (GBM-SCs), hypoxia activates a coordinated inflammatory response, evidencing an invasive and migratory phenotype.

**Methods:**

From each of 10 human solid glioblastomas, clinically and histopathologically characterized, we obtained three surgical samples taken from the center and the periphery of the tumor, and from adjacent host normal tissue. Molecular and morphological analyses were carried out using quantitative real-time PCR and western blot (WB). GBM stem and differentiated cells were incubated under hypoxic conditions and analyzed for pro-inflammatory gene expression and for invasive/migratory behavior.

**Results:**

A panel of selected representative pro-inflammatory genes (RAGE and P2X7R, COX2, NOS2 and, PTX3) were analyzed, comparing tumor, peritumor and host normal tissues. Tumors containing leukocyte infiltrates (as assessed using CD45 immunohistochemistry) were excluded. Selected genes were overexpressed in the central regions of the tumors (i.e. in the more hypoxic areas), less expressed in peripheral regions, and poorly expressed or absent in adjacent normal host tissues. Western blot analysis confirmed that the corresponding pro-inflammatory proteins were also differently expressed. Hypoxic stem cell lines showed a clear time-dependent activation of the entire panel of pro-inflammatory genes as compared to differentiated tumor cells. Biological assays showed that invasive and migratory behavior was strengthened by hypoxia only in GBM stem cells.

**Conclusions:**

In human solid glioblastoma we have observed a coordinated overexpression of a panel of pro-inflammatory genes as compared to host normal tissue. We have also evidenced a similar pattern of overexpressed genes in GBM-SCs after hypoxic treatment, showing also a gain of invasive and migratory function that was lost when these stem cells differentiated. We suggest that, as has been previously described for prostatic and mammary carcinoma, in human glioblastoma acquisition of a proinflammatory phenotype may be relevant for malignant progression.

## Background

Glioblastoma multiforme (GBM) is the most common and malignant type of brain tumor in adults and is characterized on histologic examination according to hypercellularity, nuclear atypia, mitotic figures, and evidence of angiogenesis and/or necrosis. The median survival for patients with GBM tumors is 12-18 months and the majority of these patients die within two years [1-3]. The current standard of care for GBM begins with maximal safe surgical resection followed by a combination of radiotherapy (RT) with temozolomide therapy [2]. GBM is characterized by invasiveness, necrosis and angiogenesis. In particular, vascular proliferation is an important aspect of GBM and correlates with the grade and aggressiveness of the tumor [4,5]. The increased vascular proliferation is thought to depend on hypoxic conditions created by the elevated growth rate of GBM. Increasing tumor size requires that GBM tumor cells maintain a balance between adaptation to hypoxia and cell death (apoptosis and central necrosis) through activation of hypoxia-inducible transcription factor 1 (HIF-1). HIF-1 is a heterodimeric protein composed of two subunits, α and β. Under normoxia HIF-1α is degraded by the ubiquitin-proteasome system, but when the intracellular oxygen concentration drops, HIF-1α is stabilized and translocates to the nucleus where it binds to HIF-1β [6]. The HIF-1α and β dimer activates transcription of genes involved in angiogenesis, glucose transport, apoptosis resistance, metastasis, inflammation, etc [7,8]. Such activation of transcription is achieved by binding of HIF-1α to hypoxia-responsive elements (HREs) located on the promoters of target genes [9]. We have shown recently that in breast and prostate tumors, as well as in breast tumor cell lines, HIF-1α has a crucial role in regulating, either directly or indirectly, the expression of pro-inflammatory genes. The pro-inflammatory molecules we have analyzed include membrane receptors for damage associated molecular patterns (DAMPs) such as RAGE and P2X7R, inducible enzymes such as COX2 and NOS2, and acute phase proteins such as PTX3 [10,11]. Furthermore, we have demonstrated that hypoxia increases the expression of chemokine (C-X-C motif) receptor 4 (CXCR4) which, in turn, stimulates migration of tumor cells in an in vitro assay [11]. Therefore, we sought to understand if in GBM, in which hypoxic conditions are well documented and important for tumor adaptation, we could observe an up-regulation of the same pro-inflammatory molecules analyzed in other tumors. In particular, surgical resections of cerebral GBM were achieved by craniotomy and with the aid of multiple data sets of advanced neuroimaging techniques loaded and eventually merged on the neuronavigation system used during surgery. For the purpose of this study, we merged the grid of spectroscopy MRI with T1 contrast-weighed volumetric MRI on the neuronavigation workstation, and preoperatively selected in this grid the neuropathological samples to harvest. Samples were selected from within the tumor, and in nonfunctional areas from peritumor and surrounding host tissue. In this way, we were able to study the expression of pro-inflammatory molecules in three separate samples: tumor, peritumor and surrounding host tissue.

Recently, tumor stem cells have been isolated from GBMs [12]. These tumor stem cells grow as neurospheres, possess the capacity for self-renewal, and differentiate into phenotypically diverse populations of cells similar to those present in the initial GBM. Furthermore, only these tumor stem cells are able to form tumors and generate both neurons and glial cells after in vivo implantation into nude mice [13,14]. In fact, cancer stem cells have been reported to be the only tumorigenic population in GBM, their unlimited proliferative potential being required for tumor development and maintenance [15]. Therefore, in this study we sought to determine if isolated tumor stem cells from GMB express pro-inflammatory proteins and if such expression is increased by hypoxia.

In conclusion, our study demonstrates that GBMs express pro-inflammatory proteins and that such expression is almost exclusively localized in tumor tissue, and less so in peritumor or host tissue. We also demonstrate that tumor stem cells isolated from GBM and incubated under hypoxia have a similar overexpression of pro-inflammatory proteins with increased invasion and migration.

## Methods

### Patients

Ten patients affected by cerebral GBM, as confirmed by neuropathological examination, who submitted to gross total removal of the tumor with the aid of neuronavigation and intraoperative neurophysiological monitoring, were included in this study. All patients were preoperatively submitted to functional, spectroscopy and T1 contrast-weighed volumetric MRI. No histological samples were harvested in functional areas according to both preoperative neuroradiological exams and intraoperative neurophysiological monitoring. Cases in which brain shift occurred at the dura opening were not included in this study because navigation was not considered reliable and consequently samples were not harvested with the stereotactic needle. All patients gave their informed consent for the study.

### Neuronavigation procedure

A 1.5-T clinical whole-body MR imager using polarized head coil and a gradient field strength of 44 mT/m with 124 T/m/s rise time was used. Spectroscopy was acquired with spin echo sequences (TR = 2000, TE = 135 and 30 ms) and with variable matrix (according to the site of the lesion). Postprocessing of the spectroscopic data was performed using a LCModel^® ^for Linux: filtering, apodization, phase correction and curve fitting of the main cerebral metabolites: N-acetyl aspartate, creatine, choline, lactate (when present) and myo-inositol in spectra with short TE. Ratios and maps of the above-mentioned molecules were created. The spectroscopic Cho/NAA ratio was evaluated in all voxels of an axial slice predetermined during the MRI examination. A grid of voxels with the numerical value of the ratios was superimposed over the corresponding slice of the volumetric structural dataset. The final exam was reconverted in DICOM format to be imported into the neuronavigation central unit. Here, the modified spectroscopic sequence was merged with a classical sequence without the grid: once the targets have been selected in the Navigator workstation, the grid can be suppressed (modifying the blend settings) in order not to obstruct the view of the surgeon while performing the biopsy. Each sample was surgically taken from its respective target with the aid of frameless stereotaxy (based on the Navigation technology), and subsequently harvested for molecular and histological analysis.

### Isolation, culture and differentiation of glioblastoma spheres

Tumor samples were obtained in accordance with consent procedures approved by the Institutional Review Board of the Department of Neurosurgery, Catholic University, Rome, Italy. Undifferentiated glioblastoma cells were obtained as previously described [16]. Briefly, surgical specimens were washed several times in DMEM-F12 medium supplemented with high doses of penicillin/streptomycin and amphotericin B to avoid contamination. Mechanical tissue dissociation was carried out in order to obtain single cell suspensions. Recovered cells were cultured in serum-free medium containing 50 mg/ml insulin, 100 mg/ml apo-transferrin, 10 mg/mlputrescine, 0.03 μM sodium selenite, 2 mM progesterone, 0.6% glucose, 5 mM HEPES, 0.1% sodium bicarbonate, 0.4% BSA, glutamine and antibiotics, dissolved in DMEM-F12 medium (Gibco-Invitrogen) and supplemented with 20 mg/ml EGF and 10 mg/ml bFGF. Flasks non-treated for tissue culture were used to reduce cell adherence and to support cell growth as undifferentiated tumor spheres. The medium was replaced or supplemented with fresh growth factors twice a week until the cells started to grow forming floating aggregates. Cultures were expanded by mechanical dissociation of spheres into small aggregates, followed by cell re-plating in complete fresh medium.

Differentiation of tumor stem cells was achieved by seeding dissociated cells in treated flasks in DMEM-F12 supplemented with 10% FBS. Attached cells were kept in the flasks for 1 week before performing the experiments herewith reported.

### Real-time quantitative polymerase chain reaction (RT-PCR)

Samples (cells or tissue) were lysed to extract total RNA with a BioRobot EZ1 workstation (Qiagen, Milan Italy). Approximately 1 μg of RNA was reverse-transcribed using a High-Capacity cDNA Archive Kit (Applied Biosystems, Milan, Italy) following the manufacturer's instructions. Aliquots of cDNA were subjected to real time PCR in 50 μl of 1 × Universal PCR Master Mix, 0.5 μM TaqMan probe and 5 ng of cDNA. Primers and probes for HIF-1α, NF-kB, P2X7R, RAGE, NOS2, COX2, PTX3, CXCR4, VEGF, were designed using Assays on-Demand facility (Applied Biosystems). Each sample was loaded in triplicate, and negative and positive controls were included. Amplification of 18S rRNA was used as internal reference gene. PCR amplifications were performed as follows: 50°C for 2 minutes, 95°C for 10 minutes, and 40 cycles each with 95°C for 15 seconds and 60°C for 1 minute using an ABI PRISM 7000 sequence detector (Applied Biosystems). Amplification data were analyzed using Sequence Detector version 1.7 software (Applied Biosystems). Statistical analysis of real-time PCR results were done using mean normalized cycle threshold (ΔC_t_) values and the pooled standard deviation of the mean ΔC_t_. Experiments were repeated at least 3 times with different preparations.

### Immunohistochemistry

Sections were deparaffinazed and rehydrated through a graded series of xylene-ethanol and then incubated for 15 minutes in 3% hydrogen peroxide to inhibit endogenous peroxidases. Antigen retrieval was performed by boiling slides for 15 minutes in citrate buffer. Tissue sections were incubated with the primary antibody, anti-Ki67, for 1 h at room temperature. Sections were then incubated with biotinylated secondary antibody for 20 minutes at room temperature followed by 20 minutes incubation with avidin-biotin peroxidase complex (DAKO, Milan, Italy). Visualization was performed using 3,3 diaminobenzidine. Tissue sections were counterstained with hematoxylin, dehydrated and mounted.

### Western blot assay

Cells were pelleted at 700 × *g *(5 min at 4°C) and lysed in 50 μl of cell lysis buffer (20 mM Tris, pH 7.4, 100 mM NaCl, 1% Triton, 1 mM phenylmethylsulfonyl fluoride, 10 μg/ml leupeptin, 10 μg/ml aprotinin). Alternatively, fresh frozen tissues were resuspended in a suitable volume of lysis buffer and homogenized. Afterwards, lysates were clarified by centrifugation (10 min at 4°C) and the supernatant collected. Protein concentration was determined by the Bradford assay (Bio-Rad). Equivalent amounts of protein were electrophoresed on SDS-polyacrylamide gels. The gels were then electroblotted onto PVDF membranes. After blocking with 5% milk, membranes where incubated with the primary antibody overnight. Finally, the relevant protein was visualized by staining with the appropriate secondary horseradish peroxidase-labeled antibody for 1 hour followed by enhanced chemiluminescence. Densitometric scanning analysis was performed by Mac OS 9.0 (Apple Computer Int), using NIH Image 1.62 software.

### Antibodies

The following primary antibodies were used: mouse anti-Ki67 (MIB-1) (DAKO), mouse anti-HIF-1α, mouse anti-CXCR4, mouse anti-Lamin A/C (BD Bioscience, San Jose, CA), rabbit anti-NOS2, mouse anti-CD45, rabbit anti P2X7R, goat anti-RAGE, rabbit anti-NF-kB p65, (Santa Cruz Biotechnology, Santa Cruz, CA), rabbit anti-PTX3 (Alexis Biochemical, San Diego, U.S.A.), rabbit anti-COX2 (Cayman Chemical, Ann Arbor, MI), mouse anti-β-actin (Sigma-Aldrich, St Louis, MO), CD133 PE (Militenyi Biotec, Bergisch Gladbach, Germany), and glial fibrillary acific protein (GFAP) (DAKO). The following secondary antibodies were used: anti-mouse Alexa 488, (Invitrogen-Molecular Probes, Eugene, Oregon), mouse anti-rabbit HRP, goat anti-mouse HRP (Amersham Biosciences, Piscataway, NJ), and donkey anti-goat HRP (Santa Cruz).

### Hypoxia

Hypoxic conditions were achieved by incubating glioblastoma stem cells in a hypoxia chamber (Billups-Rothenberg Inc. CA) where a 1% oxygen mix was flushed in for 4 minutes according to the manufacturer's instructions.

### Flow cytometry

Tumor spheres were dissociated as single cells, washed and incubated with the PE conjugated anti-CD133 antibody. After a 45-min incubation, cells were washed and analyzed with a COULTER EPICS XL flow cytometer (Beckman Coulter, Fullerton, CA, USA).

### Invasion assay

Cell invasion and migration was measured using a 24-well cell invasion assay kit from Millipore (Billerica, MA) and following manufacturer's instructions. Briefly, an equivalent number of cells were resuspended in serum free medium containing 1% BSA, seeded in an insert and then placed inside a 24-well plate. Each well was filled with 500 μl of serum-free medium/1% BSA. The plate was incubated in normoxia or hypoxia for the indicated time in the presence or absence of recombinant human stromal derived factor-1α (SDF-1α) (Thermo Fisher Scientific, Rockford IL) at a final concentration of 100 ng/ml. Subsequently, the insert was placed in a staining solution. Non-invading cells at the top of the membrane were removed. Invading cells, from three separate experiments, at the bottom of the membrane were stained for 10 min and then lysed. OD values, proportional to the number of cells, were measured on a plate reader with a 550 nm filter (PerkinElmer, Milan Italy).

### ELISA assay on nuclear fractions

Nuclear fractions were isolated using the nuclear extraction kit from Active Motif, (Carlsbad, CA) following the manufacturer's instructions as previously described [17]. The relative amount of HIF-1α was measured using a TransAM kit from Active Motif and following the manufacturer's instructions. Briefly, equivalent amounts of protein from nuclear extracts were loaded onto a 96-well plate coated with oligonucleotides containing HIF-1α-responsive elements. After a 1-hour incubation at room temperature, the wells were washed 3 times in wash buffer and incubated 1 hour with a primary antibody at room temperature. Subsequently, the wells were washed and incubated 1 hour with HRP-conjugated secondary antibody followed by addition of a developing solution. A stop solution was added to block the reaction and color intensity was read in a plate reader with a 450 nm filter. The color intensity in each well is proportional to the amount of the transcription factor bound to the oligonucleotides.

### Immunofluorescence and confocal analysis

Differentiated GBM stem cells were grown on poly-D-lysine coated coverslips, fixed and permeabilized as described below and then incubated with anti-GFAP antibody. After a PBS wash, cells were incubated with an Alexa 488-conjugated secondary antibody. Stained cells were washed and visualized as described below.

Normoxic and hypoxic glioblastoma tumor spheres were collected by centrifugation at room temperature (100 × g for 2 min). After a PBS wash, tumor spheres were included in gelatin that was solidified in ice at 4°C for 1 h. Solid gelatin was included in OCT and 6 μm sections obtained with a cryostat. Tumor sphere sections were then fixed in 4% paraformaldehyde for 20 min, washed with PBS, permeabilized for 6 min in 0.1% Triton and then incubated for 3 hours at room temperature in the primary antibody mix (mouse anti-human HIF-1α diluted 1:100, 1% BSA, in PBS). Afterwards, sphere sections were washed 4 times with PBS and incubated for 1 h with an anti-mouse Alexa 488 fluorescent secondary antibody. Tumor spheres sections where then washed 4 times and the cover slips were mounted in 50% glycerol solution. Fluorescence intensity was visualized with a confocal microscope (Nikon Eclipse TE 2000-E). Tumor sphere sections were also stained with hematoxylin/eosin as described above. The same immunofluorescence methodology, without sectioning, was applied for GFAP analysis on differentiated GBM stem cells. Nuclei were stained with DAPI (Invitrogen, Carlsbad, CA) following the manufacturer's instructions.

### Statistical analysis

Results are expressed as mean ± standard deviation (SD) and 95% confidence intervals (95% CI). Before using parametric tests, the assumption of normality was verified using the Shapiro-Wilk W-test. Student's paired t-test was used to determine any significant differences before and after treatment. Significance was set at p ≤ 0.05. An SPSS statistical software package (SPSS Inc., Version 13.0.1 for Windows Chicago, IL, USA) was used for all statistical calculations.

## Results

### Neuronavigation system

**Figure 1:**
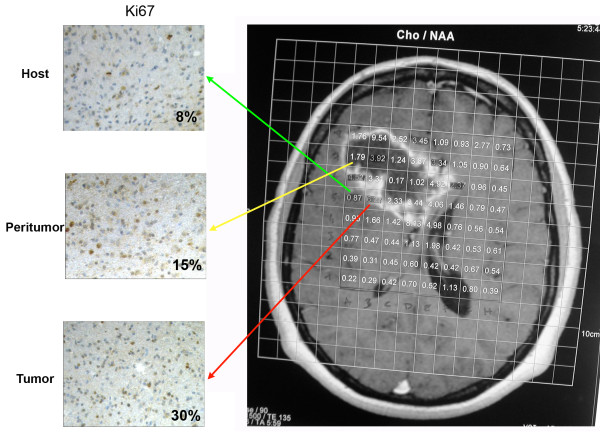
**Proliferation profiles assessed by Ki67 immunostaining in host, peritumor and tumor fractions obtained by neuronavigation**. Spectroscopic Cho/NAA ratios were evaluated in all voxels of a predetermined axial slice during the MRI examination. Voxels with low, intermediate and high Cho/NAA ratio were chosen to obtain host, peritumor and tumor fractions as described in Methods. Cell proliferation indices for each fraction were determined using immunostaining with Ki67. The percentage of Ki67-positive cells is indicated in each image. H: host tissue; P: peritumor tissue; T: tumor tissue.

Figure 1 shows evaluation of the spectroscopic Cho/NAA ratio in all voxels of a predetermined axial slice during MRI examination. The grid of voxels represents the numerical value of the ratios and has been superimposed onto the corresponding slice of the volumetric structural dataset. Each sample was surgically taken from its respective target based on the Cho/NAA ratio. In particular, the Cho/NAA ratios of the samples collected with frameless stereotaxy were considered as follows: above 1.8 for tumoral areas (T), between 0.9 and 1.8 for peritumoral areas (P), and below 0.9 for host normal areas (H). Figure 1 shows an example of the H, P and T areas chosen as well as the proliferation rate measured using the Ki67 staining. In particular, Ki67, an index of cellular proliferation, was expressed in <8% of cells of the host tissue; this percentage went up to 15% in the peritumor tissue and reached 30% in the tumor tissue (Figure 1). Importantly, these fractions with increasing Cho/NAA ratios and Ki67 expression were used throughout the study to asses changes of expression of the molecules analyzed.

### Expression and localization of HIF-1α and NF-kB in the glioblastoma tumor microenvironment

**Figure 2:**
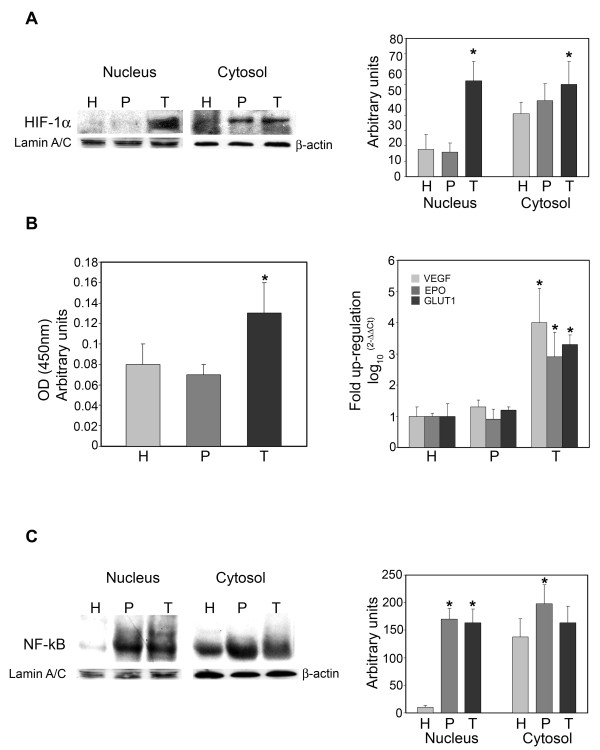
**HIF-1α and NF-kB expression in host, peritumor and tumor fractions obtained by neuronavigation**. **(A) **Host (H), peritumor (P) and tumor (T) tissues of GBM were obtained by neuronavigation. The content of HIF-1α was determined by western blotting. Values obtained from densitometric analyses of cytosolic and nuclear fractions from all samples analyzed (10) are shown. β-Actin and Lamin A/C were used as loading controls for the cytosolic and nuclear fractions, respectively. Data are presented as mean ± SD for all GBMs analyzed (n = 10). *, P < 0.05. **(B) **Left side: Host (H), peritumor (P) and tumor (T) tissue of GBM were obtained by neuronavigation. HIF-1α content was measured in the nuclear fractions by Trans-AM ELISA assay as described in Methods. Right side: VEGF, EPO and GLUT1 mRNA expression was measured in H, P and T tissues by RT-PCR. Data are presented as mean ± SD for all GBMs analyzed (n = 10). *, P < 0.05. **(C) **Host (H), peritumor (P) and tumor (T) tissues of GBM were obtained by neuronavigation. Content of the NF-kB subunit p65 was determined by western blotting. Values obtained from densitometric analysis of cytosolic and nuclear fractions from all samples analyzed (n = 10) are shown. β-Actin and Lamin A/C were used as loading controls for the cytosolic and nuclear fractions, respectively. Data are presented as mean ± SD for all GBMs analyzed (n = 10). *, P < 0.05.

Figure 2A shows expression levels of HIF-1α in nuclear and cytosolic fractions in host, peritumor and tumor tissues obtained as described in Figure 1. An increase in HIF-1α levels was observed in both nuclear and cytosolic fractions from tumor tissue, and only in the cytosolic fraction of peritumor tissue when compared to host tissue. However, only in the tumor tissue was such an increase statistically significant. A similar result was obtained by studying the binding capacity of nuclear HIF-1α by Trans-AM ELISA assay. Figure 2B shows an increase in nuclear HIF-1α bound to HRE-containing oligonucleotides in tumor versus host or peritumor tissue. No differences were observed between host and peritumor tissues. Finally, the hypoxic signature was determined by measuring mRNA expression levels for VEGF, EPO and GLUT1. Figure 2B, right side, shows that these three known target genes of HIF-1α were up-regulated only in the tumor tissue.

NF-kB is a transcription factor lying at the crossroads of inflammation, tumor transformation, response to hypoxia, etc. We, therefore, studied the expression of NF-kB in cytosolic and nuclear fractions of host, peritumor and tumor samples obtained using the neuronavigator. Figure 2C shows a significantly increased expression of NF-kB in the nuclei of peritumor and tumor samples when compared to host tissue. A significant increase in NF-kB was observed only in the cytosolic fraction of the peritumor tissue (Figure 2C).

### Pro-inflammatory gene expression in the glioblastoma tumor microenvironment

**Figure 3:**
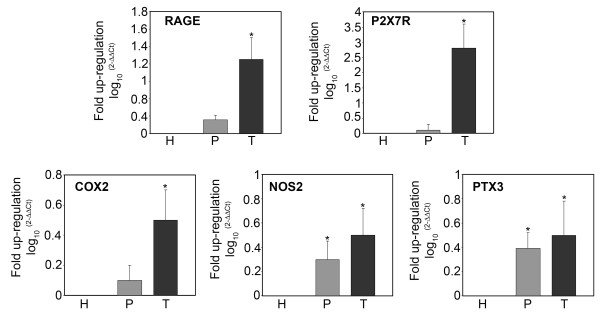
**mRNA expression levels of pro-inflammatory genes in host, peritumor and tumor fractions obtained by neuronavigation**. Host (H), peritumor (P) and tumor (T) tissues of GBM were obtained by neuronavigation. Each fraction was processed and mRNA obtained as described in Methods. mRNA expression levels for RAGE, P2X7R, COX2, NOS2 and PTX3 were determined by real time PCR. The bar graphs show fold increases in expression of the studied molecules in peritumor and tumor tissue with respect to host tissue, set at 0. Data are presented as mean ± SD for all GBMs analyzed (n = 10). *, P < 0.05.

mRNA expression of genes involved in the inflammatory response in tissue samples from host, tumor and peritumor samples of glioblastoma tumors was analyzed by RT-PCR. Figure 3 shows expression levels for the alarmin or DAMP receptors RAGE and P2X7R, for the inducible enzymes COX2 and NOS2, and for the acute phase protein PTX3. mRNA expression of the membrane receptors RAGE and P2X7R was up-regulated in tumor versus host tissue. Figure 3 also shows that mRNA expression of COX2 was up-regulated in tumor tissues, whereas no change was observed when comparing peritumor and host tissue. By contrast, NOS2 mRNA showed a pattern of increasing up-regulation from peritumor to tumor tissue compared to the host tissue. Finally, PTX3 mRNA was increasingly up-regulated from peritumor to tumor tissue in comparison to expression levels in host tissue (Figure 3).

### Pro-inflammatory protein expression in the glioblastoma tumor microenvironment

**Figure 4:**
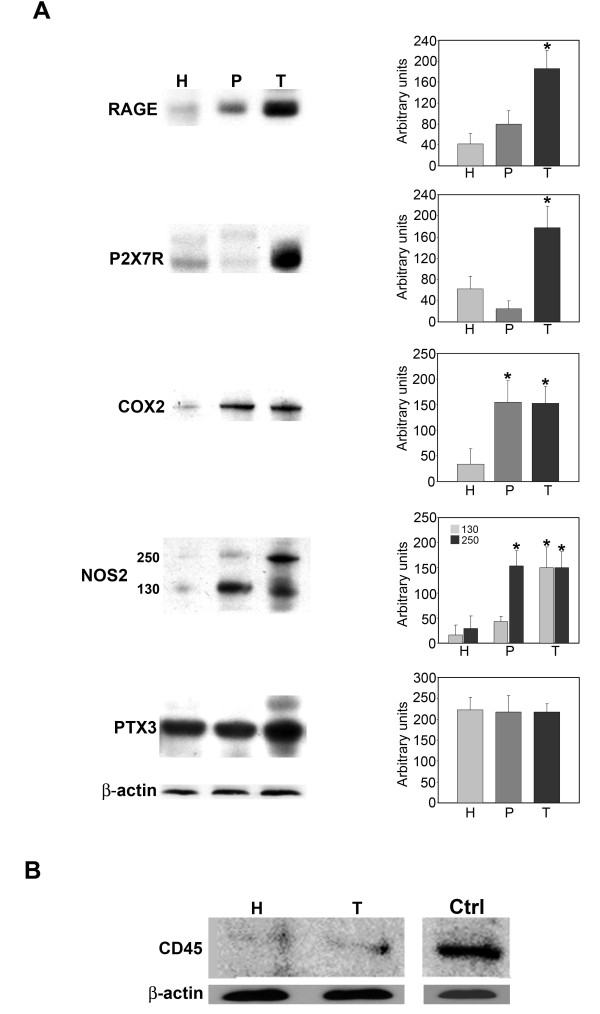
**Protein expression levels of pro-inflammatory molecules from host, peritumor and tumor fractions obtained by neuronavigation**. **(A) **Host (H), peritumor (P) and tumor (T) tissues of GBM were obtained by neuronavigation and protein expression levels of pro-inflammatory molecules were measured using western blotting. Values obtained from densitometric analysis of RAGE, P2X7R, COX2, NOS2 and PTX3 in tumor and peritumor tissues with respect to host cells from each biopsy are expressed as arbitrary units in each graph. A representative western blot for each molecule is shown on the left side. β-Actin was used as a loading control. **(B) **Host (H), peritumor (P) and tumor (T) tissues of GBM were obtained by neuronavigation. Content of the pan-leukocyte marker, CD45, was determined by SDS-PAGE and western blotting. A positive control for the antibody, represented by white cells isolated from a blood sample, is shown in the left lane.

Figure 4A shows expression levels for the pro-inflammatory proteins P2X7R, RAGE, NOS2, COX2 and PTX3. Separation of host, peritumor and tumor fractions from glioblastoma samples obtained by neuronavigator revealed an overexpression of all pro-inflammatory proteins examined (Figure 4A) in the tumor fractions. In fact, expression of the membrane receptors RAGE and P2X7R was up-regulated in tumor versus peritumor or host tissues (Figure 4A). The inducible enzymes COX2 and NOS2 were up-regulated in both tumor and peritumor tissue. However, the 250KDa form of NOS2 showed an increase only in tumor tissue (Figure 4A). The acute phase protein PTX3 did not show any differences among the three tissue fractions examined (Figure 4A). Importantly, host and tumor fractions examined in our study did not show any significant presence of leukocytes as shown by CD45 staining in figure 4B.

### Hypoxia regulates expression of pro-inflammatory genes and proteins in glioblastoma stem cells

**Figure 5:**
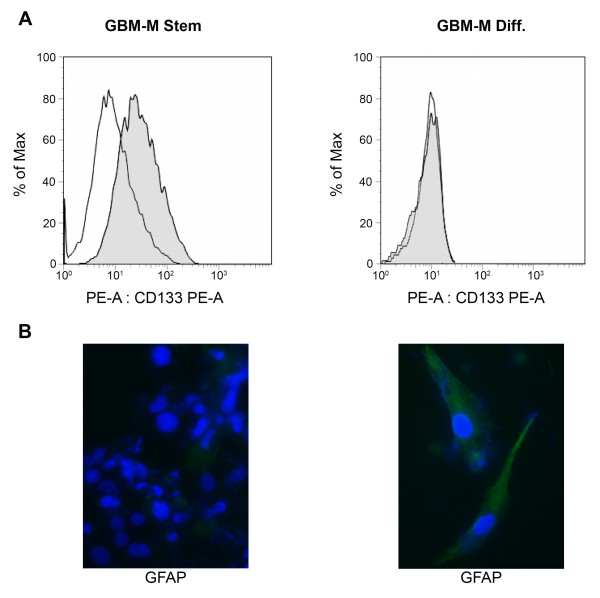
**Molecular characterization of stem and differentiated GBM cells**. **(A) **GBM stem cell spheres were stained with PE and CD133-PE and then processed by flow cytometry. **(B) **Differentiated GBM stem cells were immunostained for GFAP followed by anti-rabbit alexa 488. Nuclei were stained with DAPI. Immunofluorescence was analyzed by confocal microscopy.

Cancer stem cells are represented by those cells within a tumor that can self-renew and propagate new tumors [13,14]. In particular, cancer stem cells have been reported to be the only tumorigenic population in GBM [15]. Therefore, we isolated undifferentiated GBM cells from tumor biopsies in order to investigate if the overexpression of pro-inflammatory proteins observed in tumor fractions was also present in this population. Two different GBM stem cells populations, called GBM-M and GBM-Q, were isolated from biopsies from two separate tumors. Both GBM-M and Q grew as tumor spheres and showed similar responses to hypoxia as shown below. Furthermore, they were characterized by measuring both stem cell and differentiation-related markers. Figure 5A shows that the stem cell/progenitor marker CD133 was expressed only in GBM-M spheres. When GBM-M stem cells were differentiated for 1 week, CD133 expression was undetectable (Figure 5A). By contrast, expression of the astrocytic marker glial fibrillary acid protein (GFAP), was expressed in differentiated but not in stem GBM-M cells (Figure 5B). Similar results were obtained with the GBM-Q population (not shown).

**Figure 6:**
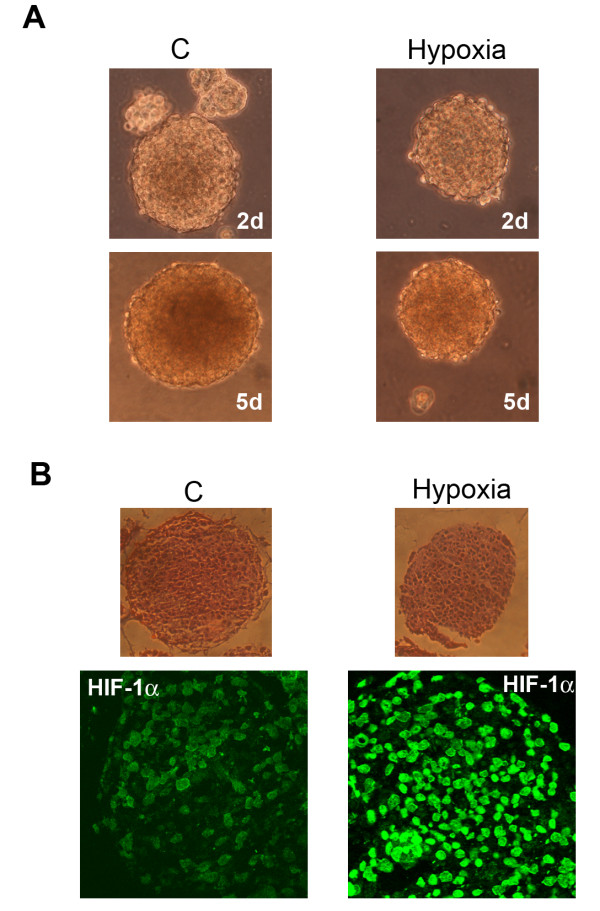
**Hypoxia influences growth and HIF-1α activation in cancer stem cells from GBM**. **(A) **GBM tumor spheres were grown either under normoxia or hypoxia for 2 or 5 days. Images of the tumor spheres were then taken using a digital camera mounted on an EclipseNet 2000 microscope. C: control normoxic cells. **(B) **GBM tumor spheres were grown either under normoxia or hypoxia for 17 h. Upper panel: Integrity of spheres was assessed in sections by hematoxylin/eosin staining. Lower panel: HIF-1α expression in sphere sections was studied by immunofluorescence as described in Methods. C: control normoxic cells.

We have previously shown that hypoxia increases expression of pro-inflammatory proteins in prostate and breast tumor cells [10,11]. Therefore, GBM stem cells were incubated under hypoxic conditions. Figure 6A shows that GBM stem cells, growing as tumor spheres, reduce their growth rate when incubated under hypoxia. However, tumor spheres survive up to 5 days of hypoxia without showing any sign of necrosis (Figure 6A). The major transcription factor activated by hypoxia is HIF-1α. Figure 6B show sections of spheres incubated either in normoxia (C) or hypoxia for 17 h. HIF-1α was expressed in the stem cells forming the GBM tumor spheres. Hypoxia incubation clearly increased HIF-1α expression and nuclear accumulation (Figure 6B).

**Figure 7:**
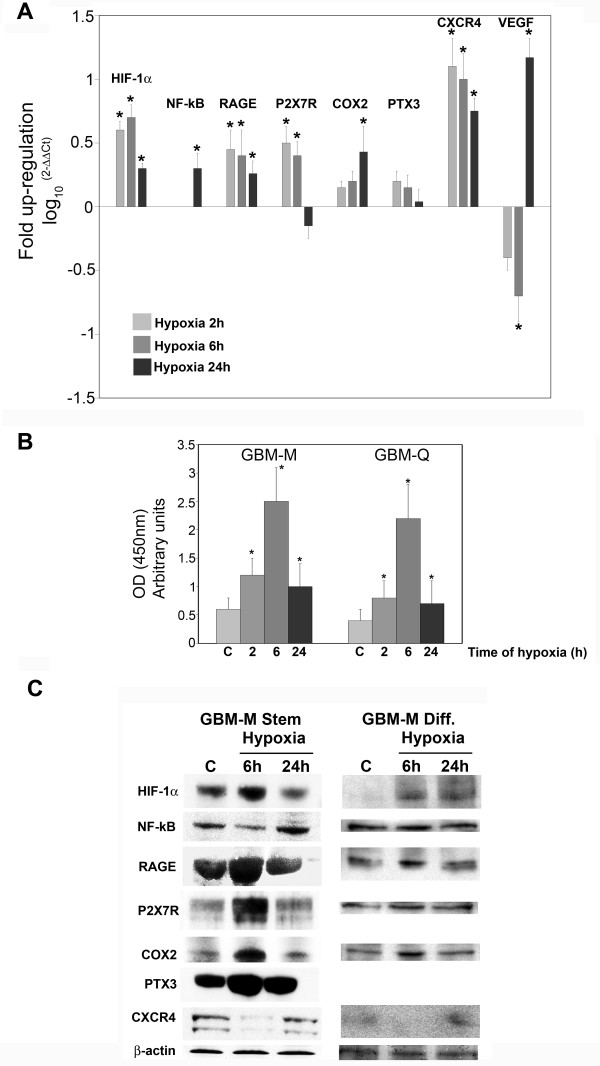
**mRNA and protein expressions of pro-inflammatory proteins in hypoxic cancer stem cells from GBM**. **(A) **GBM stem cells were incubated under hypoxic conditions for 6 and 24 h. After the times indicated, the cells were processed and mRNA obtained as described in Methods. mRNA expression levels for HIF-1α, NF-kB, RAGE, P2X7R, COX2, NOS2, PTX3, CXCR4 and VEGF were determined using real time PCR. The bar graphs show fold increases in expression of the studied molecules in cancer cells with respect to control normoxic cells, set at 0. Data are presented as mean ± SD for three separate experiments, each repeated in triplicate. *, P < 0.05. **(B) **GBM-M and -Q stem cells were incubated under normoxia or hypoxia for the times indicated. HIF-1α content was measured in nuclear fractions by Trans-AM ELISA assay as described in Methods. *, P < 0.05. **(C) **GBM-M stem and differentiated cells were incubated under hypoxic conditions for 6 or 24 h. After the times indicated, cells were processed as described in Methods. Contents of HIF-1α, NF-kB, RAGE, P2X7R, COX2, PTX3 and CXCR4 were determined by western blotting. β-Actin was used as a loading control. Blots are representative of at least three separate experiments. C: control normoxic cells.

Figure 7A shows that hypoxia influenced the expression of pro-inflammatory genes and proteins in GBM stem cells. In fact, mRNA expression for HIF-1α, NF-kB, RAGE, P2X7R, COX2, and CXCR4 was increased when stem cell spheres were incubated in hypoxia for 2, 6 and 24 h (Figure 7A). VEGF, a HIF-1α target gene, was increased after 24 h of hypoxia following a peculiar initial decrease at 2 and 6 h (Figure 7A). By contrast, PTX3 mRNA expression was not influenced by hypoxia (Figure 7A). The binding capacity of HIF-1α to HRE, measured by ELISA, showed an initial increase after 2 and 6 h of hypoxia, and a subsequent decrease at 24 h as shown in figure 7B. Both GBM-M and GBM-Q were assessed with similar results (Figure 7B). Figure 7C shows that GBM-M stem cells spheres express transcription factors such as HIF-1α and NF-kB as well as the pro-inflammatory proteins RAGE, P2X7R, COX2 and PTX3, and the chemokine (C-X-C motif) receptor 4 (CXCR4). Hypoxia increased the expression of HIF-1α at 6 h and NF-kB at 24 h. The expression of COX2, RAGE, P2X7R and PTX3 was increased after 6 h of hypoxia (Figure 7C) but showed a reduction after 24 h of hypoxia, a time point where HIF-1α expression was also significantly reduced. Finally, 24 h of hypoxia increased the expression levels of CXCR4 (Figure 7C). Interestingly, when GBM-M stem cells were differentiated for 1 week and then incubated under hypoxia, expression of the proteins examined was reduced. In fact, HIF-1α was not detected in normoxic cells as it was in the tumor spheres, but was still induced by hypoxia. NF-kB expression did not change between normoxic and hypoxic cells. The membrane receptor RAGE was expressed at lower levels and showed a low induction by hypoxia. The other membrane receptor P2X7R was present but not influenced by hypoxia. The inducible enzyme, COX2 was increased after 6 h of hypoxia, in a manner similar to what was observed in the stem cells. Finally, CXCR4 was barely detectable in differentiated cells and was not induced by hypoxia (Figure 7C).

### Hypoxia increases invasion and migration of glioblastoma stem cells

**Figure 8:**
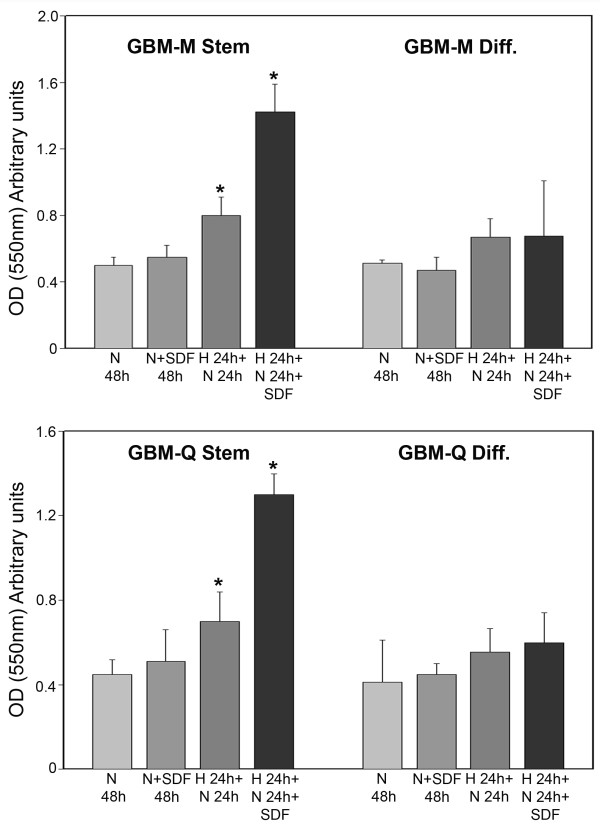
**Hypoxia increases invasion of cancer stem cells from GBM**. GBM stem and differentiated cells were incubated under normoxic conditions for 48 h. Alternatively, differentiated and stem cells were incubated under hypoxia for 24 h followed by 24 h of normoxia in the presence or absence of 100 ng/ml SDF-1α. Invading cells were stained and lysed as described in Methods. *, P < 0.05. N: normoxia. H: hypoxia. SDF: SDF-1α

Expression of membrane receptors such as CXCR4, a G_i _protein-coupled receptor for the ligand CXCL12/stromal cell-derived factor 1α (SDF-1α), has been associated with increased proliferation, invasion and migration in GBM as well as in several other tumors and tumor cell lines [18,19]. Figure 8 shows that both GBM-M and GBM-Q stem cells showed an invasive and migratory basal capacity measured after 48 h of normoxic incubation that was not increased by SDF-1α addition. Incubation of GBM stem cells under hypoxia for 24 h followed by 24 h of normoxia produced an increase in the number of invading and migrating cells. In this case, SDF-1α treatment during 24 h of normoxia incubation significantly increased the number of invading GBM stem cells (Figure 8). Finally, differentiation of GBM-M and Q stem cells significantly reduced their invasive capacities under hypoxia and hypoxia plus SDF-1α (Figure 8).

## Discussion

We have recently shown that a pro-inflammatory reparative response is activated in tumor samples of prostate and breast carcinomas in the absence of a leukocyte infiltrate [10,11]. In particular, tumor and host cells from these tumor samples, isolated by laser capture microdissection (LCMD), show an increased and coordinated expression of several pro-inflammatory proteins only in tumor cells [11]. Moreover, in the MCF-7 breast cancer cell line, we demonstrated that hypoxia stimulates coordinated expression of the same set of molecules observed in the tumor samples with an increase in cellular invasion and migration [11].

GBMs are malignant and fast growing tumors characterized by the presence of hypoxia that causes central necrosis and vascular proliferation, which are two diagnostic criteria for these tumors [20]. Recently, it has been postulated that proliferation and maintenance of GBMs are due to the presence of populations of cancer stem cells with self-renewal and tumorigenic properties [15]. In the present study we aimed to show that in GBM hypoxia and necrosis determine and maintain the expression of a coordinated pro-inflammatory response that is particularly important for tumor transformation and progression. Furthermore, we also hypothesized that such a pro-inflammatory response would be clearly present in the cancer stem cell population.

In the case of the GBMs from ten different patients, the surgical procedure using the neuronavigation system allowed us to obtain tumor, peritumor and host tissue samples. Analysis and comparison of these samples showed differential expression and subcellular accumulation of both hypoxia-regulated transcription factors HIF-1α and NF-kB and of several pro-inflammatory molecules (Figure 1, 2 and 3). None of the GBM samples we analyzed showed the presence of a leukocyte infiltrate as indicated by the absence of CD45 by western blot (Figure 4B). We acknowledge the possibility that microglia could also contribute to the pro-inflammatory genes expression we observed. In fact, by immunohistochemistry we could measure weak CD68 staining in the host, peritumor and tumor tissues examined (not shown). However, given the fact that CD68 staining is weak in tumor samples and that GBM stem cells show a pro-inflammatory gene expression, we believe that other cell types can, under hypoxia, also activate expression of pro-inflammatory genes. The presence of microglia in these samples raises also another important point, which is the importance of inflammatory cells for tumor progression. In fact, inflammatory cells, particularly macrophages, are an important part of the physiological response to the tumor. These cells are important for both tumor transformation and progression. However, we suggest that, in order to have tumor progression, another important player must be present. Such a player is represented by the expression by transformed tumor cells of pro-inflammatory proteins that allow them to survive, invade and migrate following a chemical gradient.

Both HIF-1α and NF-kB expression has been reported to be controlled by hypoxia [21]. However, the precise mechanisms and, most of all, the correlations between these two transcription factors are still obscure with some reports showing an activation of NF-kB by HIF-1α and others showing the opposite [22,23]. Our molecular analysis of tumor samples shows that HIF-1α is over-expressed and accumulates in the nucleus of the tumor fraction compared to the peritumor or host fractions (Figure 2A,B). We also observed that NF-kB expression increased in the nuclear and cytosolic fractions of peritumor tissue, and in the cytosolic fraction of tumor tissue (Figure 2C). Moreover, hypoxic incubation of cancer stem cells isolated from GBM revealed that HIF-1α and NF-kB have different time courses of activation within the first 24 h. In fact, HIF-1α expression is increased after 6 h of hypoxia and decreased at 24 h. By contrast, NF-kB expression is decreased after 6 h and increased after 24 h of hypoxia. Interestingly, the time courses of activation of pro-inflammatory proteins analyzed in hypoxic cancer stem cells were similar to that of HIF-1α, with an increase after 6 h and a decrease after 24 h. This result indicates that, at least during the first 24 h, pro-inflammatory proteins expression is influenced by hypoxia.

Analysis of pro-inflammatory phenotype in tumor samples and in cancer stem cells from GBM was conducted at the mRNA and protein levels by studying the expression of the membrane receptors RAGE and P2X7R, the inducible enzymes COX2 and NOS2, and the acute phase protein PTX3. Increased expression of RAGE and its ligand HMGB1 is usually observed in metastatic tumors and has been associated with invasion and poor prognosis [24-26]. Similarly, our results show that RAGE is clearly overexpressed in GBM tumor fractions (high Ki67 and Co/NAA) both at the mRNA and protein levels (Figure 3 and 4A). Furthermore, cancer stem cells isolated from GBM and incubated under hypoxia show an overexpression of RAGE with a time course resembling that of HIF-1α. This result indicates that, as previously shown by us in other cell lines [11], RAGE expression is controlled by hypoxia. It is worth noting that cancer stem cells have an elevated basal expression level of RAGE resembling that of the tumor fraction isolated during neuronavigation surgery, and that differentiation of GBM stem cells reduces expression of RAGE both in normoxia and in hypoxia. The purinergic receptor P2X7R has been less studied in GBMs or in glioma cell lines, and such studies have yielded the most disparate results. In fact, some reports have shown that C6 mouse glioma cells do not express P2X7R [27], whereas other reports have shown the opposite along with an important role for P2X7R in increasing tumor cell migration [28]. Furthermore, the role of P2X7R in tumor cell biology is not well understood because some results have shown a cytotoxic effect in the presence of ATP [29], whereas other results have shown a survival effect [30,31]. Finally, it has also been shown that glioma cells are more resistant to ATP toxicity when compared to normal tissue and that such resistance could contribute to invasion of tumor cells [32]. Our results, using host, peritumor and tumor fractions of GBM, have shown up-regulation of P2X7R mRNA and protein only in tumor tissue. Interestingly, this same up-regulation was observed in cancer stem cells from GBM in the presence of hypoxia. We do not know the precise role of such P2X7R up-regulation, however, by silencing P2X7R in other tumor cell lines we have observed a reduction of cell motility and invasion (Russo, personal communications). It should also be pointed out that in those reports showing ATP toxicity through P2X7R activation, cell death is not apoptotic but necrotic, with release of DAMPs that can, in turn, activate NF-kB-dependent survival pathways in the surviving tumor cells.

The inducible enzymes COX2 and NOS2 were overexpressed in peritumor and tumor tissue from GBM and, in the case of cancer stem cells, COX2 expression was increased by hypoxia. These results are in agreement with those obtained by other groups showing an up-regulation of COX2 and NOS2 that may contribute to the formation of new vessels and may represent prognostic factors [33].

PTX3 is produced and exposed on tissue cells and is considered a mediator of innate immunity and inflammation [34]. However, although it is well known that CRP is increased in tumor-bearing patients, probably because of the inflammatory reaction to the tumor presence, nobody has explored if the production of long-pentraxins and other acute phase proteins could be also due to the tumor cells themselves in response to hypoxia. In this regard, our results show that GBMs and cancer stem cells express PTX3. However, such expression is not upregulated in tumor compared to peritumor or host tissue, and is only mildly influenced by hypoxia.

Finally, we have also observed, as reported by other groups, an up-regulation of the receptor for CXC chemokines CXCR-4 in hypoxic GBM stem cells that is clearly present after 24 h of hypoxia (Figure 7C). The biological effect of this up-regulation is the increased ability of hypoxic GBM stem cells to migrate along an SDF-1α gradient. It is worth noting that differentiation of GBM stem cells strongly reduces CXCR4 expression levels as well as migration in the presence of hypoxia or hypoxia plus SDF-1α (Figure 7C and 8). These results validate our hypothesis that, compared to differentiated tumor cells, GBM cancer stem cells have a high capacity to respond to hypoxia by activating expression of pro-inflammatory genes that, in turn, confer upon these cells the ability to adapt and survive under such stressful conditions.

## Conclusions

Our study indicates that hypoxia activates a coordinated up-regulation of pro-inflammatory proteins in GBMs. Such up-regulation is particularly evident in tumor tissue when compared to peritumor and host tissue. A similar coordinated up-regulation is observed in GBM cancer stem cells under hypoxia and is strongly reduced when these cells are differentiated. Therefore, we can conclude that hypoxia due to the rapid growth observed in GBMs causes the expression of pro-inflammatory proteins that, in turn, allow tumor stem cells to activate molecular survival pathways in response to necrotic cell death.

## List of abbreviations

COX2: cyclooxygenase-2; NOS2: nitric oxide synthase-2; PTX3: pentraxin-3; RAGE: Receptor for Advanced Glycation End products; P2X7R: purinergic receptor P2X ligand-gated ion channel 7; CXCR4: chemokine (C-X-C motif) receptor 4; DAMPs: damage-associated molecular patterns; stromal derived factor-1α (SDF-1α); PE: R-phycoerythrin.

## Competing interests

The authors declare that they have no competing interests.

## Authors' contributions

MT performed the hypoxia treatments of GBM-SCs cells, performed and supervised the western blot and immunofluorescence procedures and prepared the manuscript. MDV and EM performed the real-time PCR and western blot experiments on biopsies and stem cells. AF supervised and performed all the surgical procedures. AE provided the stem cells from GBM. LP, EDS and GS performed all the necessary experiments for the revised version of the manuscript, that is: GBM differentiation, immunofluorescence, flow cytometry, ELISA and invasion assays. PS performed the confocal analysis. AS, AR and MS participated in the surgical procedures and patients' enrollment. RDM supervised the GBM stem cell isolation. MAR conceived and coordinated the project and participated in the preparation of the manuscript. All authors have read and approved the final version of this manuscript.
